# Application of High-Tech Solution for Memory Assessment in Patients With Disorders of Consciousness

**DOI:** 10.3389/fneur.2022.841095

**Published:** 2022-03-31

**Authors:** Katarzyna Kujawa, Alina Żurek, Agata Gorączko, Grzegorz Zurek

**Affiliations:** ^1^Department of Biostructure, Wroclaw University of Health and Sport Sciences, Wrocław, Poland; ^2^Neurorehabilitation Clinic, Wrocław, Poland; ^3^Institute of Psychology, University of Wroclaw, Wrocław, Poland

**Keywords:** eye tracking, memory, cognitive function, disorders of consciousness, brain damage

## Abstract

Testing cognitive function in patients after severe brain damage is a major clinical challenge. In the absence of both verbal and motor communication, tests commonly used to assess cognitive function are completely or partially undoable for disorders of consciousness patients. The study involved 12 patients with varying degrees of impaired consciousness due to brain damage, with no verbal and motor communication. Memory was assessed in study participants using oculography. Memory tasks were presented in four categories. The total percentage of correctly completed tasks obtained across the group was 39.58%. The most difficult tasks included category C.4 with tasks involving working memory. Regardless of the subjects' level of consciousness, there was no statistically significant difference in the percentage of correct responses obtained in subgroups distinguished by CRS-R score. Eye tracking technology can be successfully used in the assessment of cognitive function, particularly when eye movements are the only channel of communication in individuals after brain damage. We suggest that the cognitive functions of people after brain damage should be further analyzed using eye tracking.

## Introduction

As one of the components of cognitive function, memory is a very diverse phenomenon that is responsible for encoding information coming from the environment, consolidating the memory trace, storing it and decoding, that is, recalling the information in question, Information is encoded in the form of memory traces, the essence of which are biochemical and morphological processes. Memory traces are encoded in various brain structures in the prefrontal area, lateral temporal lobes, and hippocampus, among others (1–4).

The theoretical construct of memory has been developed and evolved over the years to the contemporary categorization of memory into long-term, short-term, and working memory. Short-term memory has been defined as temporarily available information that has limited storage time, while long-term memory has been defined as a reserve of knowledge about past events (5).

The concept of working memory is of particular interest to researchers. Baddeley and Hitch proposed a multi-component model of working memory (6), which has been developed up to modern times. The model includes: (1) the verbal working memory, (2) the visual- spatial working memory, (3) the central executive (involves the attentional control), and (4) episodic buffer (7–10). The attentional control system is responsible for manipulating, recalling, and processing non-verbal or verbal information, and the episodic buffer is a temporary system for storing, modulating, and integrating sensory information (6–8). Working memory differs from both long-term and short-term memory. It differs from long-term memory in that the information stored in working memory is not as long-lasting as long-term memory, and from short-term memory in that it involves higher-order processing and executive cognitive control not observed in short-term memory (11).

Ongoing research has brought light to the understanding of working memory. Daneman and Carpenter showed that working memory capacity (as reflected by the reading span task), correlated strongly with various comprehension tests. Similarly, in time-based resource-sharing model, working memory capacities were a product of attention that a person allocates to tasks at hand. The allocated attention and its duration accounted the likelihood of success in performing the tasks (12). Cowan argued that working memory capacity is comparable to directed or held attention information inhibition. According to him, working memory is a memory component, limited in capacity and duration, and strongly dependent on attention and other central executive processes that use stored information or interact with long-term memory (5). Working memory is an essential neural component for numerous cognitive functions, such as thinking, reasoning, decision making, and language comprehension (13). As can be seen, the current concept of working memory, while admittedly sharing similarities with short-term memory, seeks to remedy the oversimplification of its understanding by introducing the role of information manipulation (10).

Thus, contemporary understanding of memory emphasizes the role of executive processes involved in working memory (11). This is demonstrated by findings that working memory activates frontoparietal regions of the brain, including the prefrontal cortex, the cingulate cortex, and the parietal cortex. Recent studies have also indicated the role of subcortical regions such as the midbrain and the cerebellum (11).

Nevertheless, the process of remembering information does not always work properly. Memory impairment is characteristic in people after brain damage caused by injury or neurodegenerative diseases such as Alzheimer's disease (14, 15). One of the reasons for brain damage is TBI (traumatic brain injury). In a study by Dunning et al. patients with moderate to severe showed reduced cognitive function in both verbal short-term memory and verbal and visuospatial working memory when compared to control groups. In addition, time after injury and older age at injury determined cognitive decline (16), as also pointed out in a review of studies by Dikmen et al. (17). Moreover, cognitive deficits in people with neurological diseases are often multifaceted and interrelated, affecting one or more domains, not only memory but also language, perception, or abstract thinking (18, 19). Cognitive deficits also result in behavioral disturbances (20). Because of that, there is no single effective therapeutic method for people after brain damage. The interaction between different treatment modalities and interdisciplinary approach may be more effective in achieving better therapeutic outcomes in different clinical cases (21). Some literature reports that such a therapy, called multimodal therapy, is even essential in the recovery process of people after brain damage (22). Patients' recovery is possible due to the plasticity of the brain, related to its continuous adaptation to new functioning after injury and remodeling of its own structure (23). This has already been suggested by Gerard Edelman in his theory on neural Darwinism, where he emphasized that early rehabilitation of cognitive function affects supporting as many neuronal connections in the brain as possible (24, 25). At the same time, there is growing evidence that the brain can undergo significant reorganization after injury and that this reorganization can be achieved even many years after injury with appropriate late rehabilitation (26, 27). It is possible thanks to progressing knowledge in the field of neuroscience and information technology, the use of which supports the work of neuropsychologists and neurotherapists.

Nevertheless, before the patient begins neurorehabilitation, he or she undergoes diagnostics of the state of consciousness, which has a significant impact on the selection of appropriate therapeutic methods. One of the most popular scales used to assess consciousness in patients is the CRS-R (Coma Recovery Scale Revised). Patients can score a maximum of 23 on this scale. Some authors suggest, that individuals in an unresponsive wakefulness syndrome (UWS) score no more than 9 points, individuals in a minimally conscious state (MCS) score 18 points, and above that, patients qualify for eMCS (emergence from minimally conscious state) (28–30). However, the CRS-R Guidelines is more precious and determines the evaluation of the state of consciousness not only on the basis of the score value but also on the basis of the scores obtained in the individual subscales. Adequate determination of the state of consciousness is essential to determine the appropriate rehabilitation process for patients after brain damage (28–30).

Testing patients after severe brain damage is challenging, because very often patients score inadequately on tests of cognitive function as a result of impaired interaction with their environment when performing known tests such as the Mini Mental Status Examination, the Clock Drawing Test and the Montreal Cognitive Assessment. In the absence of both verbal and motor communication, these tests are completely or partially unfeasible for patients. One solution to this problem became testing patients with a binary system (yes or no answers). An example of research in a binary system conducted in 2008 is the use of the Cognitive Test for Delirium, which was designed for use with seriously ill patients. The test allows the use of non-verbal responses in the form of pointing, nodding, or raising the hand (31). In other studies, using a binary system, the way in which questions were answered was determined by the patient's degree of dysfunction; finger movements or vision, but without the use of additional eye-controlled devices (32).

Using computers to create virtual environments tailored to the sensory and motor abilities of the brain-injured patient with impaired interaction constitutes a potential solution. By doing so, contact with the community can be made possible regardless of the degree of disability (33). One form of modern information technology is brain-computer interfaces (BCIs) based on EEG signals. Based on the corresponding brainwaves, the patient's intention when performing the instructed task is determined. The responses in the form of brainwaves allow the patient to communicate without the need for behavioral responses (34, 35). An interesting example of the use of BCI changing the thinking of these patients as unable to respond to stimuli is a study conducted among patients in a state of non-reactive awakeness (formerly referred to as a vegetative state). Findings from the study indicate that some of the patients were able to repeatedly complete the task instructed to them (36).

Another popular technology is eye tracking. Eye tracking technology can be used in specific stages of the brain neurorehabilitation process, specifically when eye movements are the only channel of communication in people after brain damage. It is therefore reasonable to use eye tracking for medical purposes, for example in supporting neurological assessment of patients with severe communication barriers (37). An example of such studies is the assessment of language function in patients with impaired consciousness (38).

The literature reports that the use of eye tracking facilitates the understanding of communication signals of patients with severe motor and communication impairments. Early practice of using eye trackers can improve the quality of communication and enable more reliable patient assessments (39).

This raises the question of whether, and if so: to what extent, tasks involving different categories of memory are possible for brain-injured patients who do not communicate verbally. The answer to this question provides an opportunity to use oculography in the process of memory training in such patients. The purpose of this study was to compare the results of memory tests conducted with an eye tracker among patients with varying degrees of impaired consciousness.

## Materials and Methods

### Group Characteristics

The study group consisted of individuals between the ages of 26 and 67 who did not communicate verbally. The average age of the participants was 45.67 years. The subjects' state of consciousness was determined by the physician based on the traditional (paper) version of CRS-R. The study group was divided into three subgroups (UWS, MCS, eMCS) according to the Administration and Scoring Guidelines (28). Twelve subjects were included in the study; 7 males and 5 females whose brain damage was a consequence of different etiology: hemorrhagic stroke, ischemic stroke, trauma, and cerebral palsy (Table 1). The study group consisted of people recruited from the Palliative Care Center in Bedkowo, Poland. The inclusion criteria were: patient not undergoing hospital treatment, after completion of standard medical care, at least one functioning eyeball, consent of the patient's guardian to participate in the study. Data were collected in 2019–2020.

**Table 1:** Characteristics of the study group.

**Characteristics**						
**Subject**	**Age**	**Sex**	**Diagnosis**	**Time (months)**	**CRS-R**	**Conscious state**
P1	65	M	Hemorrhagic right-side stroke	5	16 (3/5/4/2/1/1)	MCS
P2	43	F	Ischemic stroke, both hemispheres	4	6 (2/1/2/0/0/1)	UWS
P3	42	F	Ischemic left- side stroke	4	8 (2/1/2/1/0/2)	UWS
P4	25	M	Cerebrocranial Injury	72	16 (4/5/2/2/0/3)	MCS
P5	31	M	Cerebrocranial Injury	36	18 (4/5/2/2/2/3)	eMCS
P6	40	M	Cerebrocranial Injury	36	9 (2/1/2/2/0/2)	UWS
P7	26	M	Cerebrocranial Injury	84	22 (4/5/6/2/2/3)	eMCS
P8	65	F	Hemorrhagic right-side stroke	5	20 (4/5/5/2/1/3)	eMCS
P9	67	M	Brain stem stroke	4	8 (2/1/2/1/0/2)	UWS
P10	50	F	Ischemic right-side stroke	4	4 (1/0/2/0/0/1)	UWS
P11	27	F	Cerebrocranial Injury	36	13 (3/5/2/1/0/2)	MCS
P12	67	M	Hemorrhagic right-side stroke	4	22 (4/5/6/2/2/3)	eMCS

*Time, interval of time from the critical event to the beginning of the study; P, patient; F, female; M, male; MCS, minimally conscious state; UWS, unresponsive wakefulness syndrome; eMCS, emergence from minimally conscious state*.

The study protocol received a positive opinion from the Senate Committee on Research Ethics at the Wroclaw University of Health and Sport Sciences, Poland (Approval Number: 29/2017). All participants' guardians provided written informed consent for participation and publication of this report in accordance with the guidelines established by the Declaration of Helsinki.

### Device Used in the Study

Data collection was made possible by using an eye-controlled, eye-tracking-based device that recorded data separately for each patient. Eye tracker uses invisible infrared radiation (IR) in its operation, without disturbing the patient. The device consisted of a monitor mounted on a rack with a metal boom, allowing it to be placed directly in front of the subject's face. For proper operation of the eye tracker, the first action after startup was to perform a calibration. Calibration was based on the patient's observation of a red, flickering dot with a white border, displayed in the center of the screen. After a few seconds, the started moving randomly across the screen. The system analyzed gaze fixation and dot tracking. The system reported the calibration success. If the patient was unable to fixate the eyes, the system reported that the calibration was not successful and therefore it was impossible for the patient to work with the device (40).

### Memory Evaluation

Each patient had 12 tasks to complete. The tasks involved one of four categories: memory for visual material (C.1), semantic memory (C.2), orientation to time (C.3), and working memory (C.4).

In the C.1 category tasks, three black and white pictographs were presented. Each patient had 15 s to memorize them. Time was measured by the device using a displayed stopwatch. After a time specified by the software, the pictograms flipped, changing their appearance to white squares with a question mark. The device automatically read aloud the command “find the displayed example”. At the very top of the screen, only one image was presented to be found under the corresponding question mark. Tasks in category C.2, involved choosing the appropriate pictogram from the three displayed. The example presented fit only one of them; the other two were not thematically related to the example. The tasks in this category were also shown as black outlines of objects on a white background. Category C.3; device displayed and read command: point to appropriate photo; day. Choosing from three photographs, the patient selected, for example, the time of day that was appropriate to the command heard. In category C.4, the patient was told to look closely for 5 s and to memorize three consecutively displayed images. In the last part of the task, the device displayed three sets of images, with only one set containing the correct order of the previously displayed items. Each patient worked for 1 h with the device. We observed situations when during the examination the patient would close his eyes for a moment or direct his gaze away from the monitor. The researcher tried to encourage further cooperation by calling the patient by name. If the subject continued to close their eyes, the researcher allowed the patient to rest.

**Figure 1:**
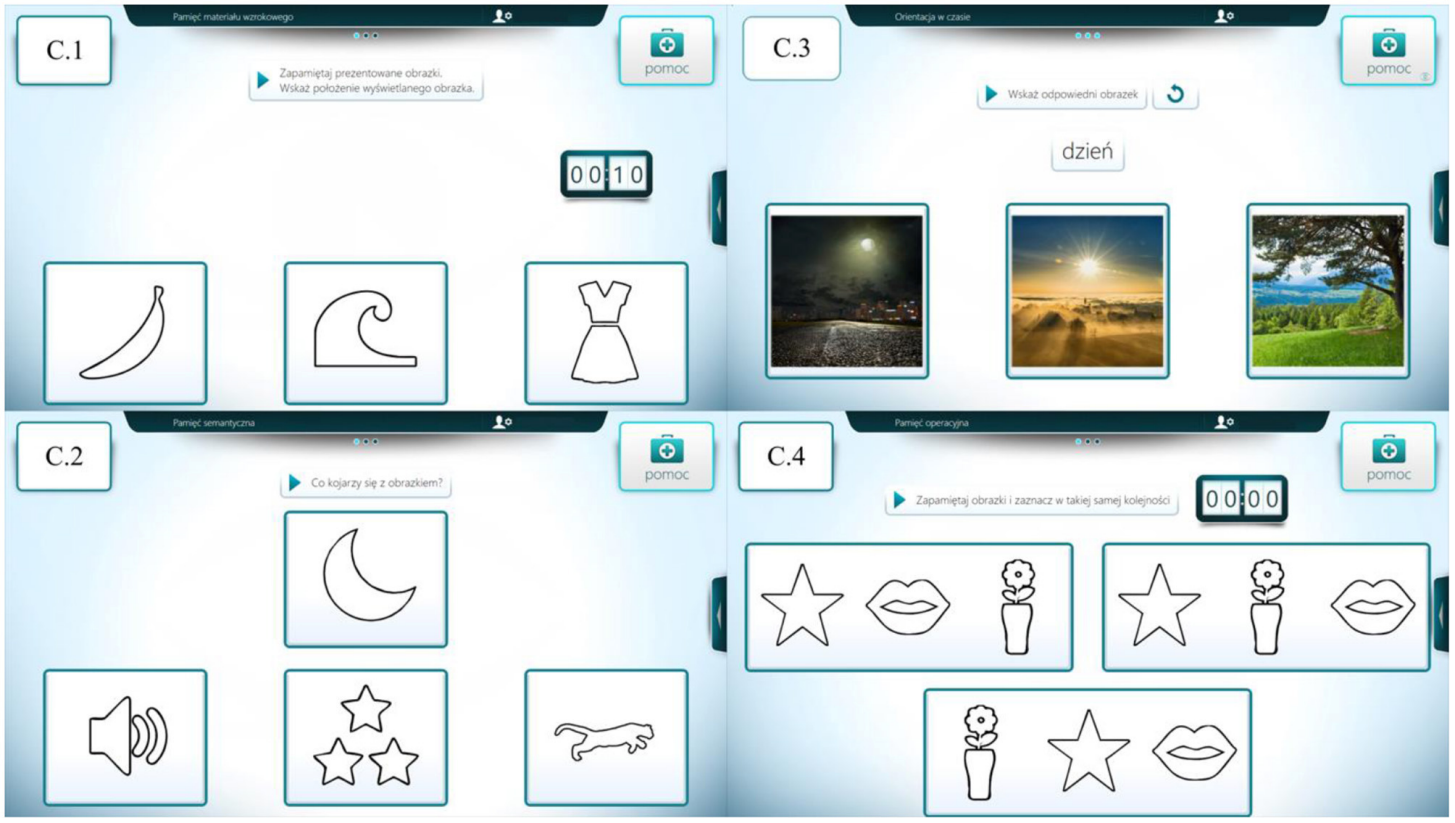
Graphical representation of the four task categories. Own source: C.1—memory of visual material, C.2—semantic memory, C.3—orientation to time, and C.4—working memory.

Images of the four task categories are shown below (Figure 1) while examples of memory tasks by category are included in Supplementary Material.

### Statistical Analyses

We performed basic statistical analyses. All analyses were performed for the entire group and for three subgroups listed by their scores on the CRS-R, providing information on their level of consciousness. We first analyzed the distributions of correct responses, then counted median values (minimum and maximum value, lower and upper quartile) and percentages of correct responses separately for subgroups and categories. In this paper, we chose to present analyses using percentages of correct responses. For this purpose, we used the Kruskal-Wallis rank-sum test to analyze the differences between the percentages of correct responses across subgroups and across the four task categories, namely for C.1, C.2, C.3, and C.4 (Table 2). Additionally Friedman's ANOVA were performed on each patient's results to verify whether it is significantly higher than random level. We found significant (non-random) differences in the distributions between the categories of tasks that patients performed: C.2 vs. C.4; C.3 vs. C.4. The package used was Statistica, ver.13.1 PL licensed to Wroclaw University of Health and Sport Sciences, Poland.

**Table 2:** Results of the study group by state of consciousness.

	**Characteristics**						
**State of consciousness**	**Subject**	**CRS-R**	**C.1**	**C.2**	**C.3**	**C.4**	**Total % of** **correct answers in** **C.1, C.2, C.3, and C.4**
			**% of correct answers**				
UWS	P10	4	33.33	66.67	0	0	25.00
	P2	6	66.67	0	66.67	0	33.33
	P3	8	66.67	0	0	0	16.67
	P9	8	33.33	100	100	66.67	75.00
	P6	9	0	66.67	0	0	16.67
Total % correct answers by UWS			40.00	46.67	33.33	13.33	33.33
MCS	P11	13	0	66.67	66.67	33.33	41.67
	P1	16	33.33	100	100	33.33	66.67
	P4	16	33.33	66.67	66.67	0	41.67
Total % correct answers by MCS			22.22	77.78	77.78	22.22	50.00
eMCS	P5	18	0	100	0	0	25.00
	P8		33.33	100	66.67	0	50.00
	P7	20	33.33	66.67	66.67	33.33	50.00
	P12	22	33.33	33.33	66.67	0	33.33
Total % correct answers by eMCS			25.00	75.00	50.00	8.33	39.58
Kruskal-Wallis test			*H* (2, *N* = 12) = 1.445481	*H* (2, *N* = 12) = 1.449412	*H* (2, *N* = 12) = 2.180139	*H* (2, *N* = 12) = 1.238426	*H* (2, *N* = 12) = 2.312218
*p*			*p* = 0.4854	*p* = 0.4845	*p* = 0.3362	*p* = 0.5384	*p* = 0.3147
Total % correct answers by whole group			30.56	63.89	50.00	13.89	39.58

*UWS, unresponsive wakefulness syndrome; MCS, minimally conscious state; eMCS, emergence from minimally conscious state; C, category*.

## Results

**Figure 2:**
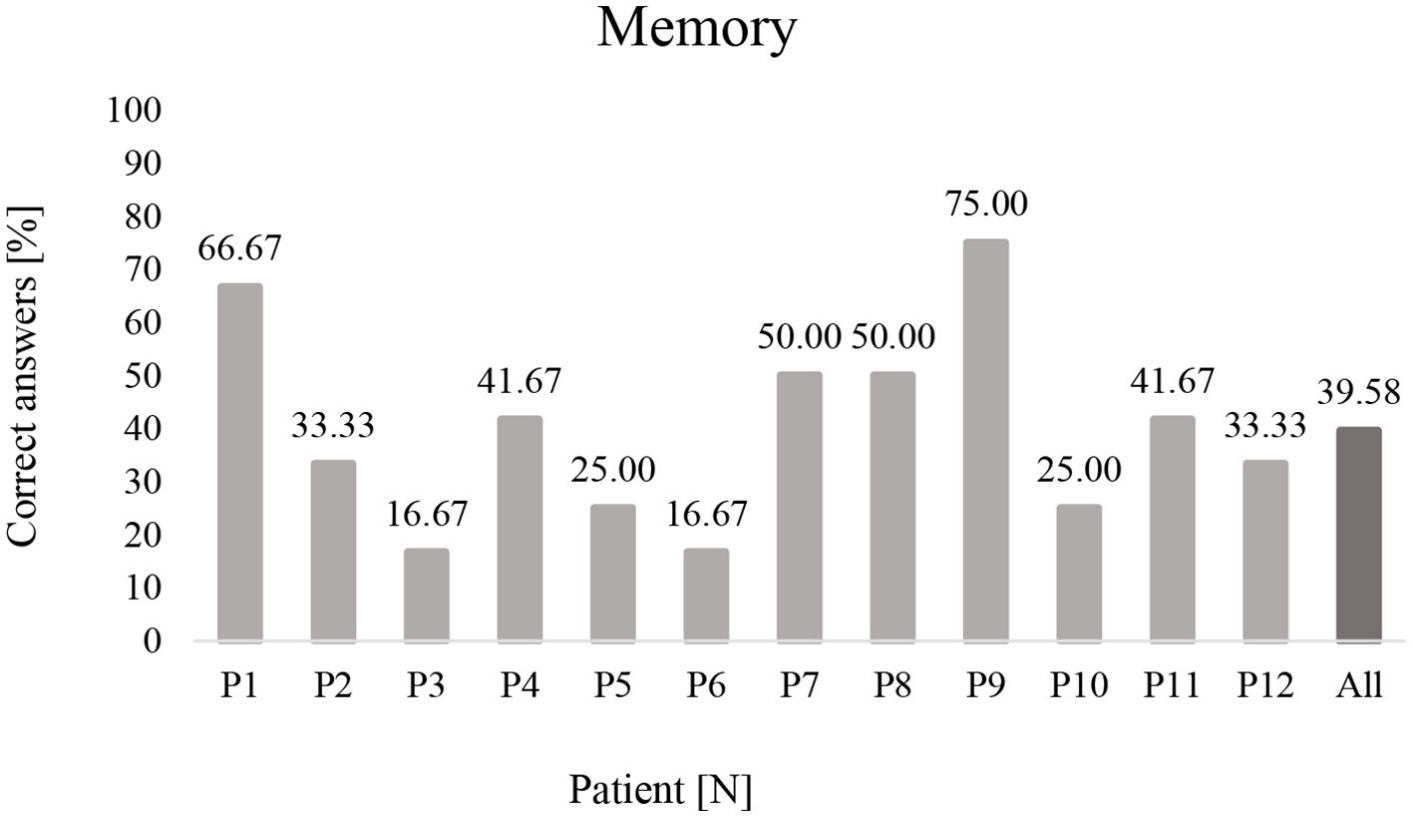
Percentage of correct answers made by patients from memory tasks (P1-P12). P, Patient.

**Figure 3:**
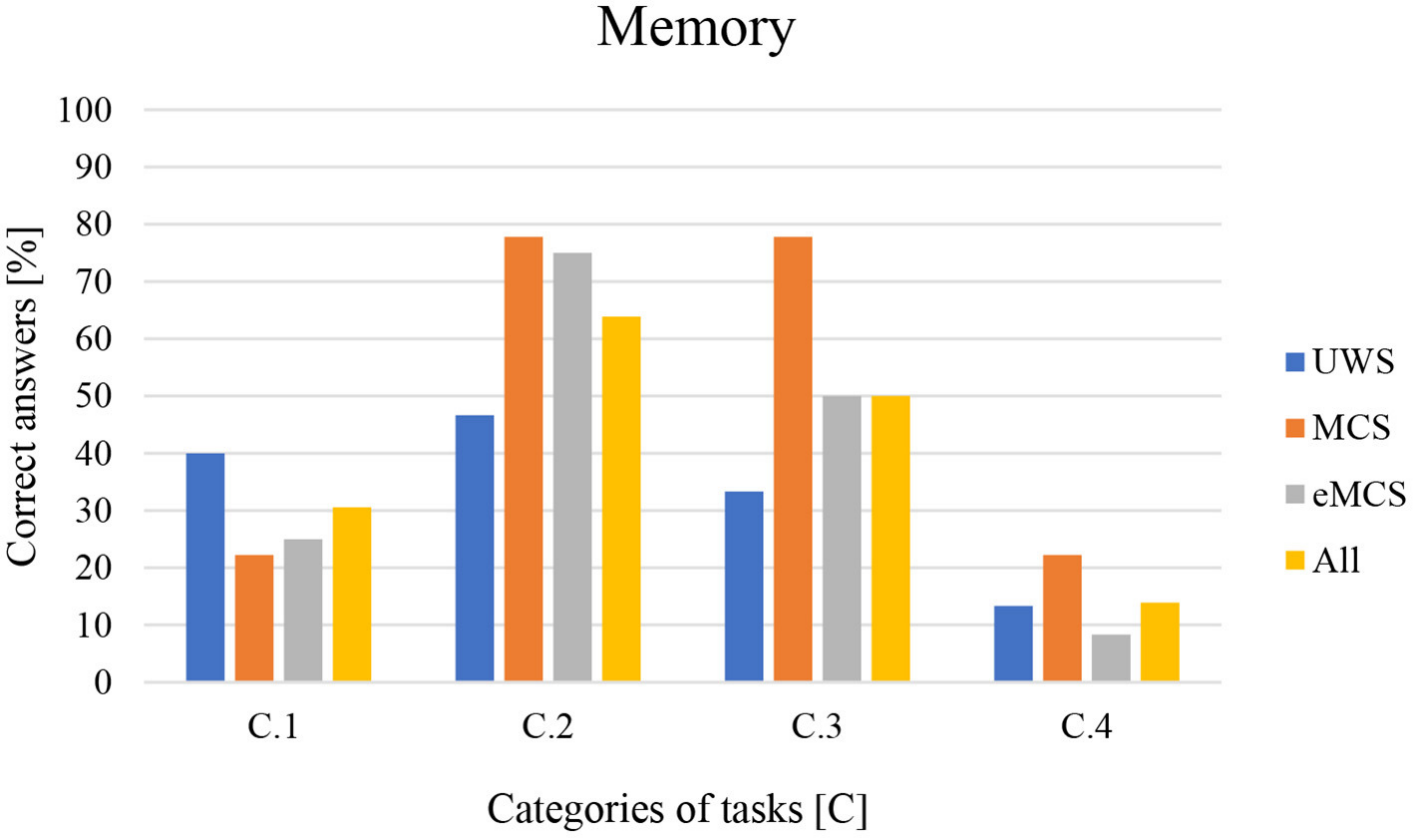
Percentage of correct answers in the tasks by C.1–C.4 category. C.1—memory for visual material, C.2—semantic memory, C.3—orientation to time, and C.4—working memory.

The results of the subjects broken down into the three subgroups of UWS, MCS, and eMCS are included in Table 2. Without specifying the categories, the highest percentage of correct responses was observed among patients diagnosed with MCS (50.00%), followed by eMCS (39.58%), and finally UWS (33.33%). On the other hand, taking into account the task categories, the highest percentage of correct answers was in category C.2 (63.89%), followed by category C.3 (50.00%), and the lowest in category C.4 (13.88%). On the other hand, when analyzing the percentage of correct answers given in the respective task categories by subjects with different state of consciousness, categories C.2 and C.3 in the MCS subgroup came to the fore with the percentage of correct indications of 77.78% each, while the lowest results were observed in category C.4 with a low percentage of correctness (8.33%) in the group with eMCS diagnosis, as shown in Figure 2. In general, the UWS had the highest scores in C.2 and the lowest in C.4, the MCS group had the highest scores in categories C.2 and C.3 and the lowest in C.1 and C.4, and the eMCS group had the highest scores in C.2 and the lowest in C.4 (Figure 3). These preliminary analyses indicate that, regardless of the subjects' level of consciousness, tasks in category C.4 involving working memory proved to be the most difficult, while tasks in category.

C.2 involving semantic memory were the easiest. Additional information was contributed by analyses using the Kruskal-Wallis rank-sum test, which showed that there were no statistically significant differences between the percentage of correct responses obtained in both categories C.1, C.2, C.3, and C.4, as well as the global score obtained in subgroups distinguished by consciousness level. This means that regardless of the consciousness level of the respondents, the percentage of correct answers is similar.

Analysis of individual patient outcomes also provides valuable information. Only four patients were able to complete at least half of the tasks (P9, P1, P7, and P8). Of particular note are the highest scores obtained by P9 and P1, qualified for UWS and MCS, respectively, with CRS-R scores of 8 and 16, respectively. With significantly different scores on the CRS-R, they achieved similarly high completion rates of 75.00 and 66.67%, respectively (a 1 point difference in raw score). As can be seen, different states of consciousness do not determine a significant difference in eye tracker test results.

## Discussion

Brain damage can lead to impaired consciousness (41), i.e., coma, UWS or MCS, among others. Coma survivors of severe TBI often suffer from long-term disability that is mainly related to cognitive deficits (42). Patients with the UWS do not communicate with their surroundings and show reflex behaviors, such as spontaneous eye opening or breathing, but without signs of awareness of themselves or their surroundings (19, 43, 44). However, the four patients with UWS in our study were able to make little contact, typically performing <1/3 of the tasks presented. One hypothesis to explain these results may be that misdiagnosis is relatively common among patients with low CRS-R scores (45). Some studies indicate that misdiagnosis rate in this group of patients exceeds 40% (46). In addition, there have been recent reports of patients who show cognitive motor dissociation—CMD (34). CMD is characteristic of patients with impaired consciousness who show neuroimaging evidence of consciousness but no detectable command execution behavior. Thus, performing CRS-R testing in such patients ultimately classifies them as UWS. This should be taken into consideration despite the fact that the CRS-R is now the recommended diagnostic scale in many countries for the evaluation of patients with disorders of consciousness (DOC) who suffered from brain damage (47, 48). Our study appears to confirm previous literature reports (49), indicating that there may be residual capacity for normal information processing in the brain damage. Furthermore, available literature suggests that brain activation patterns indicative of consciousness can be found in ~10% of patients with unresponsive wakefulness syndrome (50). The ability to process information in our UWS patients was probably sufficient enough to allow them to perform some of the tasks. At the same time, our one study patient with the same diagnosis (UWS), achieved the highest correctness rate (at ¾) among the study subjects. Such a high correctness rate of the completed tasks suggests a higher level of consciousness demonstrated by the patient, while a low score obtained by him in the CRS-R may indicate a temporary decrease in responsiveness during the conducted test.

It is worth noting the relatively high scores obtained by MCS patients, who had a task completion rate of 50.00%; this is higher than that in eMCS patients (the percentage of correct answers was 39.58%). The observed discrepancies in outcomes between the MCS and eMCS groups may be related to the observed reduction in emotional state resulting either from longing for loved ones (in the eMCS group) or are due to other factors, the identification of which requires further detailed observation and analysis. This is particularly evident in P5 (25.00%) and P12 (33.33%). Similar conclusions are drawn from other studies conducted, which also indicate a significant role of emotional state for the effectiveness of cognitive-behavioral therapy for patients after acquired brain injury (ABI). Because patients not only have to come to terms with the traumatic event, but also with the biological and psychological changes and numerous losses associated with ABI, they very often struggle with anxiety and depression which affects cognition, mood and motivation, making rehabilitation difficult (51, 52).

Respondents found category C.4, which contains tasks involving working memory, to be the most difficult tasks. This is important information for continued recovery, because it tells us that in the long term, if functional recovery were to occur, such patients may have difficulty with current tasks in daily life, because of their limited ability to use working memory in completing tasks. This conclusion is also supported by the results of other studies suggesting that severe TBI is associated with a reduction in resources within the central executive of working memory. Working memory limitations are likely related to impaired activation of the brain's executive networks due to diffuse axonal damage. These deficits have consequences that impair activities of daily living (39). Another study on a group of 37 patients undergoing neuropsychological testing to assess various cognitive functions showed severe deficits in rapid processing, divided attention, working memory, executive function, and long- term memory (53).

It is also worth noting the global percentage of correct answers obtained by all patients when performing the tasks (39.58%). Such a value may be more a result of poor eye control through weakness of the muscles controlling them, and less to do with difficulty in understanding the task due to cognitive limitations. The weaker control of eye movements in brain-injured subjects was supported by a study by Cifu et al. (54) which found that less accurate tracking of moving targets could be observed in patients who simultaneously had lower saccade amplitudes and longer durations of eye movement from one point to another.

One of the first clinical signs distinguishing MSC from UWS is eye movements occurring in direct response to moving stimuli (55). Eye movements can improve with proper training. This is indicated by a study conducted in a group of 12 post-TBI subjects, in whom eye movement was assessed before and after oculomotor training (OMT), which involved, e.g., fixation and saccadic movements. After OMT, there was a significant reduction in horizontal fixation error, while saccadic gain increased in both the horizontal and vertical planes. These results suggest improved rhythmicity, accuracy, and sequentiality of saccades after OMT (56). Having considered the foregoing, patients after TBI may benefit from solving cognitive function tasks with the help of oculography, as it is also a type of oculomotor training. These benefits occur regardless of the fact that it has not been clearly established whether fixation or saccade abnormalities in TBI are due to post-traumatic symptoms, ocular motor damage, or cognitive impairment (57). However, taking into account the foregoing considerations and the observations made during our own study, we believe that the limitation of ocular motor control was the factor determining the patients' ability to perform the task.

Although our study was based on a limited number of participants in a specific context, we have shown that patients have the most difficulty with tasks based on short-term memory. We suggest that the cognitive functions of people after brain damage should be further analyzed using eye tracking. Modern technology shows that solving various cognitive tasks leads to the emergence of brain activity states that can be precisely identified and even used to control devices external to the brain like BCIs (58). Further research is therefore needed to expand our knowledge of the frequency and duration of oculomotor training and improve its effectiveness. This will help understand what neurorehabilitation outcomes to expect when working with patients after brain damage. In the context of reports on patients with cognitive motor dissociation, it is also important to observe patients' UWS more precisely in order to compare the results obtained by patients in the traditional assessment of their state of consciousness and their ability to cooperate with eye-tracking devices.

## Limitations

There are some limitations to the study that need to be taken into account. One of them involved performing the CRS-R scale assessment once. Because behavioral fluctuations in patients may affect diagnostic accuracy, the number of assessments performed on the CRS-R may influence the clinical diagnosis of patients with chronic disorders of consciousness.

We suggest that more than one test be performed in a short interval (e.g., 2 weeks) in each patient with impaired consciousness to reduce misdiagnoses.

It also seems that it would be valuable to repeat the memory study in quick succession. This would reduce the risk of error in the assessment of memory status by averaging it and would make this result more independent of the patient's emotional state.

## Conclusions

Testing memory in people with impaired consciousness provides a better understanding of the brain function in this group of patients. The study demonstrated that memory impairment in individuals in the UWS, MCS, and eMCS groups primarily involved working memory. Innovative approaches to patients after brain damage can significantly expand our current understanding of therapy and influence the variety of therapeutic interventions and, in particular, the appropriate adaptation to the functional abilities of the patient with communication and motor disorders. In conclusion, therapy for cognitive function including memory should be introduced in brain-injured patients regardless of the degree of impairment of consciousness using oculography.

## Data Availability Statement

The raw data supporting the conclusions of this article will be made available by the authors, without undue reservation.

## Ethics Statement

The studies involving human participants were reviewed and approved by the Senate Committee on Research Ethics at the Wroclaw University of Health and Sport Sciences (Consent No. 29/2017). All participants' supervisors, or the guardians of the patients/participants, provided written informed consent for participation and publication of this report in accordance with the guidelines established by the Declaration of Helsinki.

## Author Contributions

Material preparation, data collection, and analysis were performed by KK, AŻ, AG, and GZ. The first draft of the manuscript was written by KK, AŻ, and GZ. AG, AŻ, and GZ contributed substantially to the interpretation of the results, provided critical feedback, and revised the manuscript. All authors contributed in review and editing of the manuscript, approved its final version, and contributed to the study conception and design.

## Conflict of Interest

The authors declare that the research was conducted in the absence of any commercial or financial relationships that could be construed as a potential conflict of interest.

## Publisher's Note

All claims expressed in this article are solely those of the authors and do not necessarily represent those of their affiliated organizations, or those of the publisher, the editors and the reviewers. Any product that may be evaluated in this article, or claim that may be made by its manufacturer, is not guaranteed or endorsed by the publisher.

## Supplementary Material

Supplementary Table 1 Memory for visual material (C.1), semantic memory (C.2), orientation to time (C.3), and working memory (C.4).
