# Supplementary material for: Application of High-Tech Solution for Memory Assessment in Patients With Disorders of Consciousness
Source: Front Neurol. 2022 Mar 31;13:841095. doi: 10.3389/fneur.2022.841095 (PMC9008141; doi:10.3389/fneur.2022.841095)
Supplement: Supplementary Table 1 — Memory for visual material (C.1), semantic memory (C.2), orientation to time (C.3), and working memory (C.4). [file Table_1.DOCX]

Table 1. Set of 12 tasks presented to participants on the screen of the device

| **Type of task (category)** | **Task read aloud by the device** | **Content of the task displayed on the monitor** | **Correct answer** |
| --- | --- | --- | --- |
| C.1a | Memorize the presented images  Indicate the position of the displayed image | There are 3 images displayed at the bottom of the screen:  ice cream, trapeze, lamp,  After 15 seconds, the images turn into blank squares with a question mark.  **Task**: point to the image with a trapeze using your eyes | The middle question mark under which the image of the trapeze is hidden |
| C.1b |  | There are 3 images displayed at the bottom of the screen:  ladder, chocolate, ball  After 15 seconds, the images turn into blank squares with a question mark.  **Task**: point to the image with a ball using your eyes | The last question mark under which the image of a ball is hidden |
| C.1c |  | There are 3 images displayed at the bottom of the screen:  building, train station, wolf,  After 15 seconds, the images turn into blank squares with a question mark.  **Task**: point to the image of a train station using your eyes | The middle question mark under which the image of the train station is hidden |
| C.2a | What are your associations with the image? | An image is presented at the top of the screen:  knife  3 images are displayed at the bottom of the screen:  helmet, camel, fork  **Task**: point to the correct image using your eyes | Image: Fork |
| C.2b |  | An image is presented at the top of the screen:  A person sitting on a dental chair  3 images are displayed at the bottom of the screen:  joint, teeth, sponge  **Task**: point to the correct image using your eyes | Image: Teeth |
| C.2c |  | An image is presented at the top of the screen:  A person watching TV  3 images are displayed at the bottom of the screen:  TV, lemon, mouse  **Task**: point to the correct image using your eyes | Image: TV |
| C.3a | Point to the correct image | In the upper part of the screen, there is an expression presented: summer  There are photos presented at the bottom of the screen:   - a child sledding in the winter - a child playing with a ball in the water in the summer   **Task**: point to the correct image using your eyes | Photo: a child playing with a ball in the water in the summer |
| C.3b |  | In the upper part of the screen, there is an expression presented: day  There are photos presented at the bottom of the screen:   - evening - night - day   **Task**: point to the correct image using your eyes | Photo: summer |
| C.3c |  | In the upper part of the screen, there is an expression presented: past  There are photos presented at the bottom of the screen:   - mammoth - car   **Task**: point to the correct image using your eyes | Photo: mammoth |
| C.4a | Memorize the images and mark them in the same order | Image appear consecutively in the middle of the screen for 5 seconds:  phone, moon, sausage  Three sets of images are then displayed:   - moon, sausage, phone - phone, moon, sausage - sausage, phone, moon   **Task**: identify the sequence of images displayed in the order presented at the beginning | Set: phone, moon, sausage |
| C.4b |  | Image appear consecutively in the middle of the screen for 5 seconds:  mustache, lipstick, syringe  Three sets of images are then displayed:   - mustache, lipstick, syringe - mustache, syringe, lipstick - lipstick, mustache, syringe   **Task**: identify the sequence of images displayed in the order presented at the beginning | Set: mustache, lipstick, syringe |
| C.4c |  | Image appear consecutively in the middle of the screen for 5 seconds:  tie, beard, mountains  Three sets of images are then displayed:   - beard, tie, mountains - mountains, beard, tie - tie, beard, mountains   **Task**: identify the sequence of images displayed in the order presented at the beginning | Set: tie, beard, mountains |
